# Fabrication of polyoxometalate-modified palladium–nickel/reduced graphene oxide alloy catalysts for enhanced oxygen reduction reaction activity

**DOI:** 10.1039/d1ra06936e

**Published:** 2021-12-07

**Authors:** Fereshteh Dehghani Sanij, Prabhuraj Balakrishnan, Huaneng Su, Lindiwe Khotseng, Qian Xu

**Affiliations:** Institute for Energy Research, Jiangsu University Zhenjiang 212013 China xuqian@ujs.edu.cn; Department of Chemistry, University of the Western Cape Cape Town 7535 South Africa

## Abstract

Designing advanced nanocatalysts for effectively catalyzing the oxygen reduction reaction (ORR) is of great importance for practical applications of direct methanol fuel cells (DMFCs). In this work, the reduced graphene oxide (rGO)-supported palladium–nickel (Pd–Ni/rGO) alloy modified by the novel polyoxometalate (POM) with Keggin structure (Pd–Ni/rGO-POM) is efficiently fabricated *via* an impregnation technique. The physical characterizations such as X-ray diffraction (XRD), Fourier transform infrared spectroscopy (FT-IR), Raman spectroscopy, inductively coupled plasma optical emission spectroscopy (ICP-OES), field emission scanning electron microscopy coupled with energy dispersive X-ray spectroscopy (FESEM-EDX), and transmission electron microscopy (TEM) are utilized to confirm the structure, morphology, and chemical composition of the fabricated samples. The XRD results verify the formation of the POM-modified Pd_8_Ni_2_/rGO alloy electro-catalyst with the face-centered-cubic (fcc) structure and average crystallite size of 5.54 nm. The electro-catalytic activities of the nanocatalysts towards ORR in alkaline conditions are evaluated by cyclic voltammetry (CV), rotating disk electrode (RDE), and chronoamperometry (CA) analyses. The synthesized Pd_8_Ni_2_/rGO-POM nanomaterial shows remarkably greater ORR catalytic activity and better methanol resistance compared with the Pd_8_Ni_2_/rGO and Pd/rGO electro-catalysts. The promoted ORR activity of the Pd_8_Ni_2_/rGO-POM sample is attributed to the alloying of Pd and Ni components, the uniform scattering of Pd–Ni nanoparticles on rGO, and the alloyed catalyst being modified with POM. Moreover, these findings demonstrate that the resultant Pd_8_Ni_2_/rGO-POM material is attractive as a suitable and cost-effective cathodic catalyst for DMFCs, in which the decorated POMs play a vital role for the enhancement in the catalytic abilities of the nanocatalyst.

## Abstract 2

A novel nanocatalyst, polyoxometalate-modified palladium–nickel/reduced graphene oxide (Pd_8_Ni_2_/rGO-POM), is prepared and served as an effective ORR nanomaterial in alkaline media.

## Graphical abstract

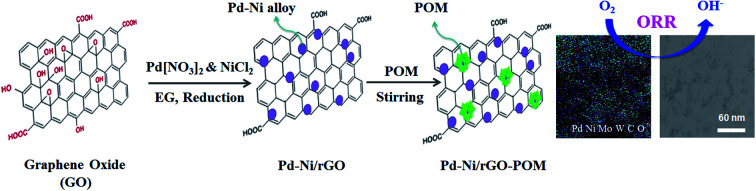

## 1. Introduction

Direct methanol fuel cells (DMFCs) have aroused great interest for energy conversion and storage in recent years due to the marked benefits, including high efficiency, simple construction, ease of charging, and non-polluting nature.^1–3^ Nonetheless, the practical development of DMFC technologies is hampered by some challenges such as the exorbitant price of platinum (Pt) electro-catalysts, slow kinetics of the oxygen reduction reaction (ORR), and inevitable crossover of methanol through the membrane.^4–6^ Hence, developing cost-effective, efficient, and methanol-resistant catalysts for the ORR is of tremendous significance.

In this context, palladium (Pd)-containing electro-catalysts, as attractive potential substitutes, have been actively explored for oxygen reduction in DMFC systems since Pd nanomaterials possess comparatively higher ORR activity compared with that of Pt electro-catalyst, but they are considerably cheaper than platinum.^7,8^ Besides, to promote the ORR performance of nanoparticulated Pd catalysts, diverse alloyed materials, alternative substrates, and modified nanocatalysts have been developed.^9–12^ With the continuing growth of nanotechnologies, various investigations have reported that Pd-based alloy materials are becoming appealing nanocatalysts. A variety of alloyed materials like PdCu,^13^ PdFe,^8,14,15^ PdAg,^16^ PdCo,^17,18^ and PdAu^19^ have been investigated on account of their greater catalytic activity and cost-effectiveness. Pd-containing bimetallic alloyed catalysts, especially Pd–Ni alloys, indicate better electro-catalytic capabilities compared with monometallic systems.^20,21^

On the other hand, to enhance the electro-catalytic ability and support the nanocatalysts, different kinds of carbon substrates such as carbon black,^10^ carbon nanotubes (CNTs),^22,23^ and graphite^24^ have been employed when utilizing Pd or Pd alloy nanoparticles for the oxygen reduction. Among them, reduced graphene oxide (rGO), as a suitable electro-catalyst substrate, has been widely exploited owing to high chemical stability along with striking electron transport properties.^25–27^

Recently, polyoxometalates (POMs) have also been integrated with nanocatalysts to increase their ORR catalytic activity. POMs, which have triggered substantial attention to be employed in electro-catalysis,^28^ energy conversion and storage systems,^29,30^ medicine,^31,32^ and material science,^33^ are the nanoscale high-valent transition metal–oxygen anionic cluster compounds with distinctive molecular structures and fascinating physicochemical properties.^34,35^ POMs have the ability to function as proton acceptors and conductors, which makes the ORR process easier.^36^ In addition, POMs reveal prominent capabilities for reducing hydrogen peroxide to water, which is the rate-controlling stage in oxygen reduction catalysis.^37,38^ In particular, the Keggin-type POMs can be readily adsorbed on Pd-based catalyst surface, and they are able to efficiently enhance the oxygen reduction activity by facilitating the charge transfer during the ORR process. Such POM modification of the Pd-based nanocatalysts would offer benefits like improved stability of the electro-catalysts and remarkable activity for ORR.^39^

The nanocatalysts created by depositing POMs on carbonous materials are more prospective than pure POMs since this enhances the low conductivity of POMs and promotes their catalytic performance. Among the carbon materials, graphene-based nanomaterials have been recognized as the most desirable carriers for POMs because they have rich channels and pores for the diffusion of oxygen in the oxygen reduction electro-catalysis procedures. For instance, Liu *et al.* synthesized innovative ternary Ag NPs@POM/rGO hybrids by a green and simple method. Interestingly, owing to the synergistic effects of Ag nanoparticles, rGO, and POM, the synthesized ternary nanohybrids revealed exceptional electro-catalytic performances towards oxygen reduction through a direct 4e^−^ procedure.^40^ In another study, Li *et al.* reported the preparation of Pd/POM/rGO nanocomposite with the help of POM for ORR. Their results confirmed the consistent dispersion of the nanosized Pd particles on the rGO surfaces. Porous nanostructures and excellent conductivity, which can be caused by the interactions between rGO and POMs, seem to be favorable for improving the permeation of reactants. The fabricated nanomaterial demonstrated superior electro-catalytic ability and better methanol resistance as compared to other studied samples.^39^ Inspired by the previous studies, the fabrication of novel POM-modified Pd alloyed nanoparticles/rGO appears highly promising.

Despite the benefits of applying decorated POMs on the Pt electro-catalysts, the impacts of POM modification of Pd-based alloy nanomaterials on catalysis of oxygen reduction reaction have been described rarely, presumably because the Pt and Pt-based catalysts are currently in their real implementation in fuel cell technologies, while the Pd and POMs are still in their ongoing investigation phase. Thus, the design of POM-modified Pd–Ni/rGO material as an efficient nanocatalyst for oxygen reduction in DMFC systems is a challenging task. In the present study, POM-modified Pd_8_Ni_2_/rGO (henceforth designated as Pd_8_Ni_2_/rGO-POM) was fabricated *via* the facile impregnation method, in which well-dispersed Pd–Ni nanoparticles were first prepared and anchored on the rGO surface, and then POM was decorated on the Pd_8_Ni_2_/rGO sample. These nanocatalysts were structurally characterized using various physicochemical techniques. Furthermore, their electro-catalytic behaviors towards ORR were compared with Pd_8_Ni_2_/rGO and Pd/rGO to assess the influences of rGO and POM over the prepared Pd_8_Ni_2_/rGO-POM nanocatalysts. The electro-chemical results revealed that Pd_8_Ni_2_/rGO-POM has the highest activity and stability for oxygen reduction comparing to the other two catalysts. Hence, it is worthwhile to employ such an outstanding Pd_8_Ni_2_/rGO-POM nanomaterial as the cathode electro-catalyst for DMFCs.

## 2. Experimental

### 2.1. Materials

Graphite powder, Nafion solution (5 wt%), disodium hydrogen phosphate (Na_2_HPO_4_), potassium permanganate (KMnO_4_), sodium tungstate dihydrate (Na_2_WO_4_·2H_2_O), and sodium molybdate dihydrate (Na_2_MoO_4_·2H_2_O) were supplied from Sigma-Aldrich. Methanol (MeOH), hydrochloric acid (HCl), nickel(ii) chloride hexahydrate (NiCl_2_·6H_2_O), sulfuric acid (H_2_SO_4_), palladium(ii) nitrate dihydrate (Pd[NO_3_]_2_·2H_2_O), ethylene glycol (EG), nitric acid (HNO_3_), sodium hydroxide (NaOH), diethyl ether (Et_2_O), isopropyl alcohol (IPA), hydrogen peroxide (H_2_O_2_), and sodium nitrate (NaNO_3_) were purchased from Merck. Commercial Pt/C product (20 wt%, from ETECK) and other chemical materials involved were utilized as received. All chemicals in this investigation were directly used with no more pretreatments. Deionized (DI) water was employed to dilute and dissolve chemical reagents during the experiments.

### 2.2. Preparation of Pd–Ni/rGO electro-catalysts

The modified Hummers' method^41,42^ was utilized to produce GO from graphite powder. An impregnation technique was used for the synthesis of the binary Pd–Ni alloy materials with 10 wt% metal content, as detailed in our earlier work.^21^ In short, the GO was initially suspended in a mixture of IPA/H_2_O (10 ml, *V*_IPA_/*V*_H2O_ = 1 : 1) and exfoliated by ultrasonic processing for half an hour to generate a uniform dispersion. Based on the atomic proportions of samples, certain amounts of Pd (0.033 mmol, Pd[NO_3_]_2_·2H_2_O) and Ni (0.019 mmol, NiCl_2_·6H_2_O) precursors were then added into the support solution under continuous stirring for 15 min till the suspension was well blended. To complete the reduction of metallic ions, after adjusting the pH value to 10–12 by means of 1.0 M NaOH and then subsequently adding EG (60 ml) as a reducing factor, the mentioned mixture was stirred at 120 °C under the blow of argon (Ar) with continuous reflux for 16 h. At this step, the bimetallic Pd–Ni alloyed nanoparticles supported on rGO were constructed because of the simultaneous reduction of metallic ions and GO. The achieved solution was naturally cooled down to room temperature (RT), and the solid powders were separated using centrifugation and rinsed three times with DI water for eliminating any residual precursors along with maintaining the pH about 7. After then, the final product was kept in the oven at 70 °C for overnight drying and denoted by Pd_8_Ni_2_/rGO. For comparative investigation in oxygen reduction, Pd/rGO catalyst was synthesized adopting a similar approach through mixing a dispersion of GO with appropriate amounts of Pd precursor (0.041 mmol, Pd[NO_3_]_2_·2H_2_O). All the other preparation steps were maintained the same as those needed for fabricating binary alloyed Pd–Ni/rGO catalyst.

### 2.3. Preparation of Pd–Ni/rGO-POM electro-catalyst

The H_3_[PMo_4_W_8_O_40_] was prepared through the described process by Huixiong *et al.*^43^ The Pd–Ni/rGO-POM electro-catalyst was fabricated as follows: 0.02 g of Pd–Ni/rGO nanocatalyst was immersed in 2.58 × 10^−3^ M POM solution.

**Scheme 1:**
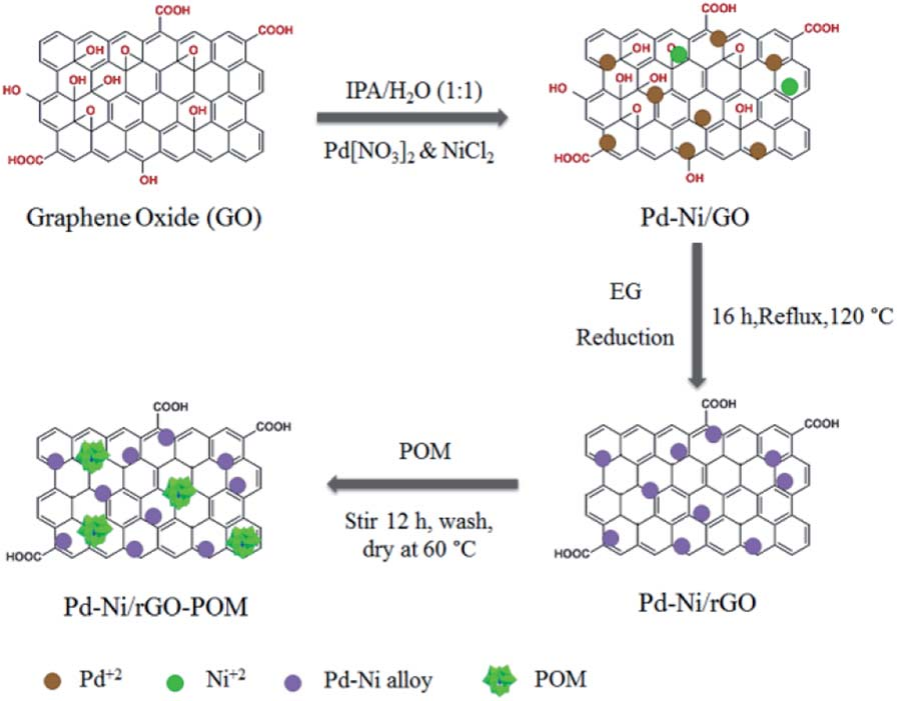
Schematic illustration of the synthetic process for the Pd_8_Ni_2_/rGO-POM electro-catalyst.

Afterwards, this mixture (pH ∼ 6.5) was kept stirring for 12 h. Ultimately, after the resulting product was filtered, rinsed, and dried for 24 h in a vacuum oven at 60 °C, the Pd–Ni/rGO-POM sample was acquired. Scheme 1 demonstrates the synthetic process of the Pd_8_Ni_2_/rGO-POM electro-catalyst.

### 2.4. Physical characterization

The crystal nanostructures of fabricated materials were achieved with recording X-ray diffraction (XRD) scans by filtered Co Kα radiation on a Phillips X'Pert MPD diffractometer. The Fourier transform infrared (FTIR) measurements were carried out on a Nicolet AVATAR 370 spectrometer within the spectral range of 600 and 4000 cm^−1^. The Raman spectra were achieved using an Almega Thermo Nicolet dispersive Raman spectrometer with a 532 nm laser excitation source. The inductively coupled plasma optical emission spectroscopy (ICP-OES) was performed on a PerkinElmer Optima 8000 instrument. The field emission scanning electron microscopy (FESEM) and energy-dispersive X-ray spectroscopy (EDX) together with the corresponding elemental mapping were conducted on a TESCAN Mira 3-XMU. The transmission electron microscopy (TEM) images were collected on a Phillips model CM200 microscope.

### 2.5. Electro-chemical characterization

All the electro-chemical ORR studies of prepared nanocatalysts were performed on a three-electrode based system in 0.1 M KOH or 0.1 M KOH/1 M MeOH solutions saturated with O_2_ as electrolyte. In this research, the catalytic tests were conducted at 25 °C, and a fabricated nanomaterial loaded glassy carbon (GC, area of 0.031415 cm^2^) was served as a working electrode. An Ag/AgCl (saturated with KCl) and a 1 cm^2^ Pt plate were utilized as reference and auxiliary electrodes, respectively.

The suspension of as-obtained sample powders (1 mg) in a mixed solvent (1 mL, IPA and H_2_O = 2 : 1) and Nafion ionomer (1 mL, 0.18 mg mL^−1^) was formed by a 20 min sonication. Before coating the electro-catalyst samples on the surface of the electrode for electro-chemical measurements, the GC electrode was polished successively by alumina slurries and cleaned ultrasonically in DI water. The specified amount of respective electro-catalyst suspension was then coated evenly on the working area of cleaned GC electrodes. Eventually, after air drying, the metal loading of studied nanomaterial on the active surface of each electrode was set to 0.1 mg cm^−2^.

Cyclic voltammetry (CV) curves were recorded in the potential range of −1.2 to 0.2 V *vs.* Ag/AgCl at the scanning rate of 50 mV s^−1^. Linear sweep voltammetry (LSV) experiments were conducted on rotating disk electrodes (RDE) with various rotating per minute (rpm) velocities (400 to 2000 rpm) at the scan rate of 5 mV s^−1^. Chronoamperometry (CA) measurements were accomplished at a fixed potential of −0.4 V *vs.* Ag/AgCl.

## 3. Result and discussion

### 3.1. Physical characterization of electro-catalysts

**Fig. 1:**
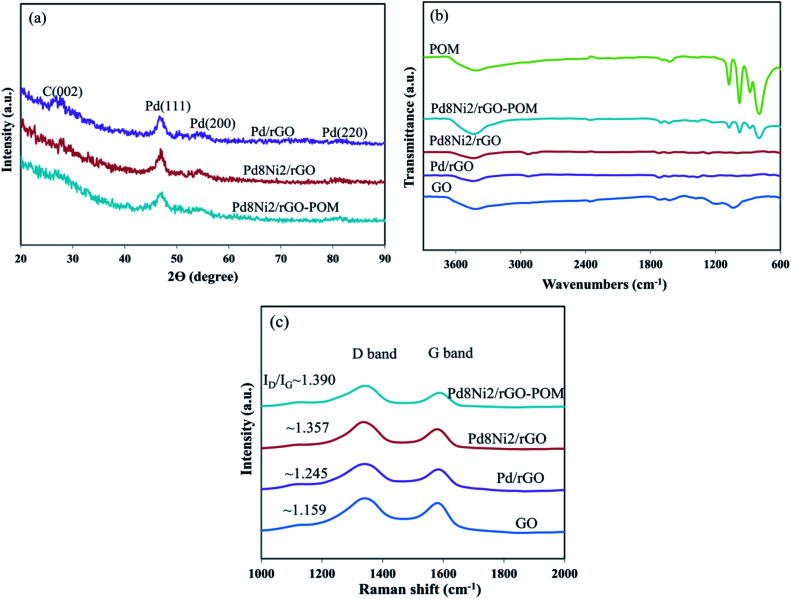
XRD patterns (a), FTIR spectra (b), and Raman spectra (c) for different nanomaterials.

The structures of the synthesized materials were examined using XRD analyses. As indicated in the XRD patterns of the studied nanocatalysts (Fig. 1a), the first peak at around 28° is assigned to the (002) plane of rGO support.^44^ The Pd/rGO catalyst presented the typical peaks of a crystalline face-centered-cubic (fcc) Pd, which are related to the (111), (200), and (220) planes (JCPDS-46-1043). For Pd_8_Ni_2_/rGO and Pd_8_Ni_2_/rGO-POM, the Pd diffraction peaks showed a slight shifting relative to Pd/rGO, demonstrating that alloying of Ni with Pd was conducted.^45^ Notably, no clear characteristic peaks of individual Ni elements can be observed, further signifying that the Pd–Ni alloy systems are successfully generated.^46^ Besides, the Pd_8_Ni_2_/rGO-POM nanomaterial does not exhibit any peaks of POM in the XRD patterns, implying that the POM structures are present in the dispersed form instead of the crystalline form.^47,48^ The average crystallite sizes of the Pd/rGO, Pd_8_Ni_2_/rGO, and Pd_8_Ni_2_/rGO-POM catalysts were evaluated by Scherrer's equation^49^ utilizing Pd(111) reflection, and the obtained results are depicted in Table 1. As shown in Table 1, the incorporation of Ni into Pd can inhibit the growth of Pd–Ni nanocrystals, resulting in smaller crystallite sizes.^50,51^ The calculated average crystallite sizes of the Pd_8_Ni_2_/rGO and Pd_8_Ni_2_/rGO-POM electro-catalysts are both 5.54 nm, which will be confirmed by the TEM results later.

**Table 1:** Physical characterization results of the Pd/rGO, Pd_8_Ni_2_/rGO, and Pd_8_Ni_2_/rGO-POM electro-catalysts

Electro-catalyst	Composition					Crystallite size (nm), XRD	Particle size (nm), TEM
	ICP (wt%)				EDX		
	Pd content	Ni content	Mo content	W content	Pd/Ni		
Pd/rGO	9.85	—	—	—	—	7.66	7.57
Pd_8_Ni_2_/rGO	7.59	2.25	—	—	4.36	5.54	5.38
Pd_8_Ni_2_/rGO-POM	7.79	2.20	3.72	7.67	4.68	5.54	5.38

The FT-IR spectra of synthesized nanostructures are indicated in Fig. 1b. As displayed in Fig. 1b, the FT-IR spectrum of GO presents the characteristic peaks at 3407, 1711, 1624, 1351, and 1041 cm^−1^, which were associated with the O–H, C=O, C=C, C–OH, and C–O stretching vibrations, respectively.^52,53^ In the case of prepared rGO-supported catalysts, the decreased intensities of characteristic peaks originating from oxygen functionalities signify the efficient reduction of GO to rGO.^54^ The absorption bands at 1072 cm^−1^ (*ν*_as_(P–O)), 977 cm^−1^ (*ν*_as_(metal=O)), 879 cm^−1^ (*ν*_as_(metal–O–metal)), and 786 cm^−1^ (*ν*_as_(metal–O–metal)) belonging to the main characteristic peaks of POM, were depicted in the FT-IR spectral scan of POM.^43^ These observations approve that the POM has the Keggin structure. The FT-IR spectrum of Pd_8_Ni_2_/rGO-POM exhibited that the POM structure has remained in the catalyst and verified the existence of POM in the prepared nanomaterial (Fig. 1b).

Fig. 1c illustrates the Raman spectra of GO, Pd/rGO, Pd_8_Ni_2_/rGO, and Pd_8_Ni_2_/rGO-POM materials, in which the D-band (∼1343 cm^−1^) is associated with the intervention of the sp^2^ hybridized carbon atoms and the G-band (∼1583 cm^−1^) is assigned to the sp^2^ hybridized carbon atoms within the hexagonal graphite networks. The peak intensity ratio of these two bands, *I*_D_/*I*_G_, is generally utilized to assess the size of sp^2^ domains and disorder degrees in graphite materials.^55,56^ The differences in values of *I*_D_/*I*_G_ can be due to the incremented defects in graphene nanosheets, which originate from the electronic relations between carbon atoms and metallic nanoparticles (Fig. 1c). In comparison to GO, the *I*_D_/*I*_G_ value incremented for all as-prepared nanomaterials, which demonstrates that the GO nanosheets have been efficiently reduced to form graphene.^57^

**Fig. 2:**
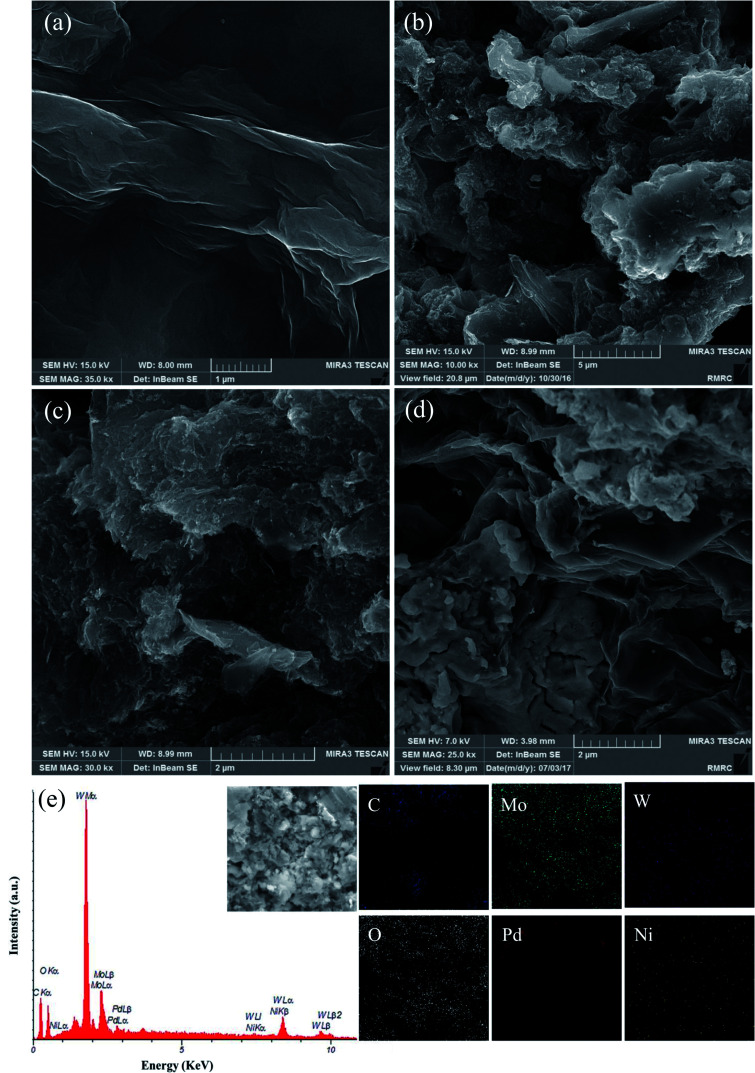
FESEM images of the GO (a) Pd/rGO (b), Pd_8_Ni_2_/rGO (c), Pd_8_Ni_2_/rGO-POM (d), EDX spectrum and elemental mappings of C, Mo, W, O, Pd, and Ni (e) for the Pd_8_Ni_2_/rGO-POM.

The morphologies of GO, Pd/rGO, Pd_8_Ni_2_/rGO, and Pd_8_Ni_2_/rGO-POM samples were examined using FESEM (Fig. 2). FESEM image of GO shows thin layered structures with smooth surfaces and slightly crumpled edge planes (Fig. 2a). As seen in Fig. 2b and c, the nanosized Pd and Pd_8_Ni_2_ particles were homogeneously dispersed onto the rGO surface. After the decoration of POM on the Pd_8_Ni_2_/rGO catalyst, the surfaces of the rGO substrate were also covered by POM particles (Fig. 2d). The elemental mapping images of Pd_8_Ni_2_/rGO-POM for C, Mo, W, O, Pd, and Ni elements are shown in Fig. 2e. The simultaneous existence of constituent elements in Pd_8_Ni_2_/rGO-POM presents the effective preparation of this catalyst. Moreover, the elemental mappings confirm the even dispersity of all the components in the Pd_8_Ni_2_/rGO-POM material.

EDX and ICP-OES measurements were carried out to analyze the composition of the synthesized nanomaterials (Table 1). The characteristic peaks of C, O, Mo, W, Pd, and Ni were identified in the EDX spectrum of the Pd_8_Ni_2_/rGO-POM sample (Fig. 2e). The obtained outcomes specified that the Pd_8_Ni_2_/rGO material is actually decorated by POM because just this POM structure contains Mo and W components. Moreover, the actual metal (Ni and Pd) loadings of the nanocatalysts have been determined using ICP-OES analysis to be around 10 wt%.

**Fig. 3:**
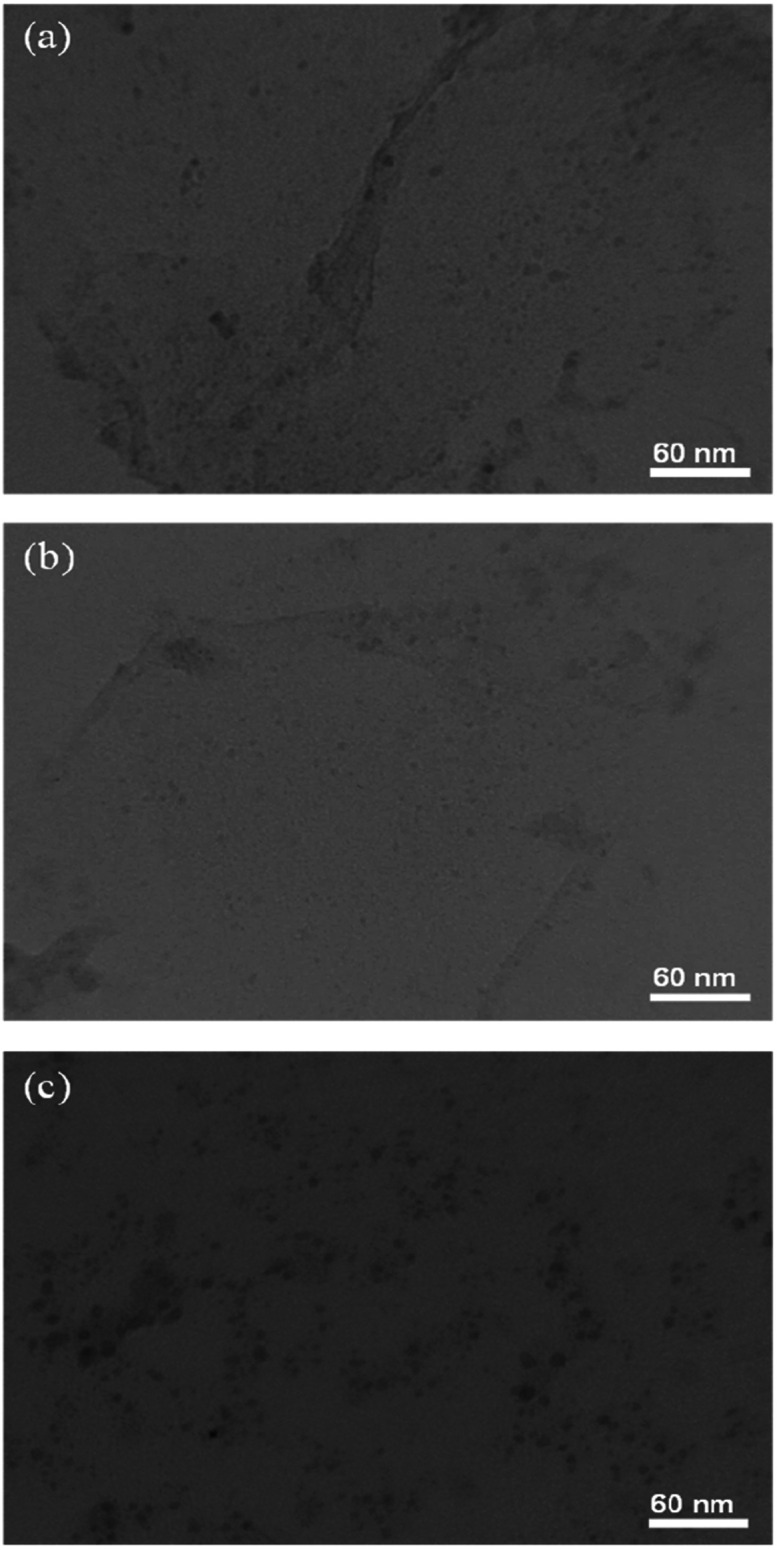
TEM images of the Pd/rGO (a), Pd_8_Ni_2_/rGO (b), and Pd_8_Ni_2_/rGO-POM (c).

TEM images for Pd/rGO, Pd_8_Ni_2_/rGO, and Pd_8_Ni_2_/rGO-POM samples are presented in Fig. 3. Noticeably, there are several spherically shaped nanoparticles over the surface of substrates, evidencing the effective preparation of metallic nanoparticulated catalysts on rGO. Besides, it is noteworthy to mention that the shape of the Pd_8_Ni_2_/rGO nanomaterial has no marked difference after decorated using POM.

Fig. 3a–c shows that the Pd and Pd_8_Ni_2_ nanoparticles are evenly distributed on the rGO substrate in the Pd/rGO, Pd_8_Ni_2_/rGO, and Pd_8_Ni_2_/rGO-POM materials. As observed from XRD results, the deposition of Pd and Pd–Ni on the rGO supports can lead to the generation of uniformly dispersed metal nanoparticles with smaller particle sizes compared to other previously reported catalysts on different substrates.^58–60^ These observations unambiguously imply that the rGO substrate is more suitable for anchoring well-dispersed Pd nanoparticles. The sizes of Pd–Ni nanoparticles are similar for both alloyed catalysts (Table 1), indicating that nanoparticle size influences on the catalytic abilities over the POM decorated Pd_8_Ni_2_/rGO electro-catalyst can be ignored in the current research. The results of TEM and XRD analyses reveal that the POMs decorated over the surface of Pd_8_Ni_2_/rGO nanocatalyst hardly affect its structural characteristics with the least remarkable aggregation of the Pd–Ni nanoparticles.

### 3.2. Electro-chemical properties of electro-catalysts

#### 3.2.1. Cyclic voltammetry (CV) and linear sweep voltammetry (LSV)

**Fig. 4:**
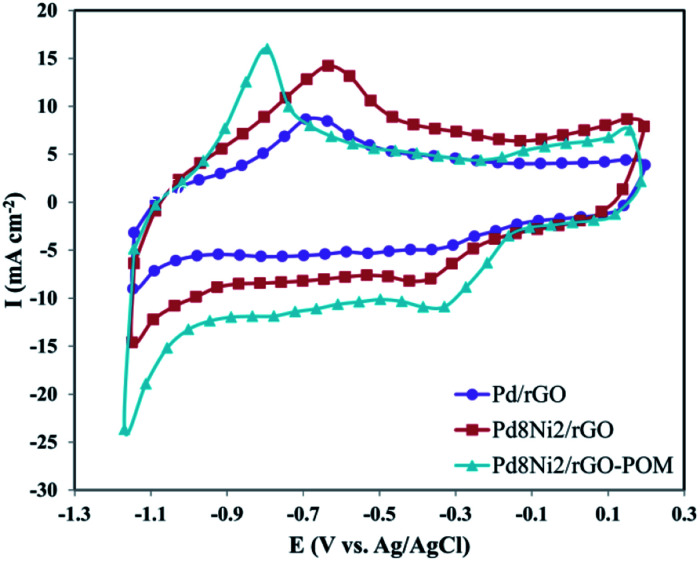
The CV curves of the Pd/rGO, Pd_8_Ni_2_/rGO, and Pd_8_Ni_2_/rGO-POM electro-catalysts in O_2_-saturated 0.1 M KOH solution at a scan rate of 50 mV s^−1^.

To evaluate the electro-chemical behaviors of the synthesized Pd/rGO, Pd_8_Ni_2_/rGO, and Pd_8_Ni_2_/rGO-POM nanocatalysts for ORR, the CV measurements were performed in O_2_-saturated 0.1 M KOH electrolytes at a scan rate of 50 mV s^−1^ (Fig. 4). As indicated in Fig. 4, the peak potentials of ORR are noticed for each of the nanocatalysts. In comparison with the other two catalysts, Pd_8_Ni_2_/rGO-POM exhibited more positive peak potential and greater current density towards oxygen reduction. This reveals a significant improvement in the oxygen reduction activities for Pd_8_Ni_2_/rGO-POM relative to the Pd_8_Ni_2_/rGO and Pd/rGO electro-catalysts. The improved ORR performance of the Pd_8_Ni_2_/rGO-POM catalyst can be connected with beneficial synergistic effects of the alloyed Pd–Ni nanoparticles and rGO as well as the promotion of charge transfer between bimetallic nanoparticles and POM-modified surface of rGO substrate.

**Fig. 5:**
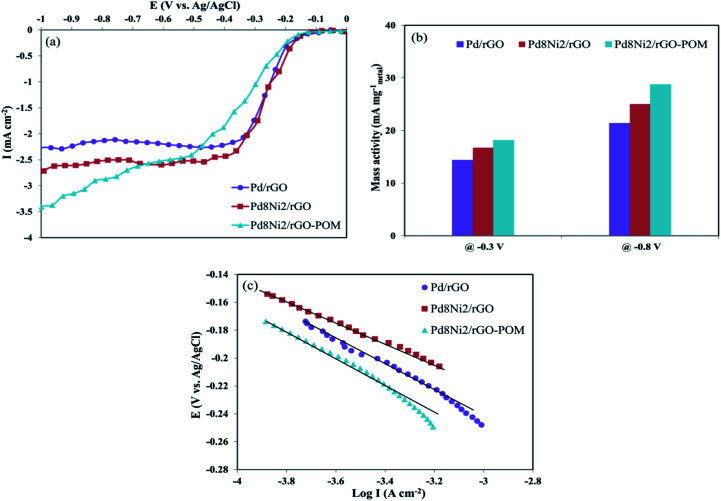
The LSV curves of the Pd/rGO, Pd_8_Ni_2_/rGO, and Pd_8_Ni_2_/rGO-POM electro-catalysts in O_2_-saturated 0.1 M KOH solution at a scan rate of 5 mV s^−1^ and a rotating speed of 1600 rpm (a). Comparative mass activity of these electro-catalysts at −0.3 V and −0.8 V (b). Tafel plots of all electro-catalysts (c).

The LSV technique was applied to assess the intrinsic electret-catalytical activities of Pd/rGO, Pd_8_Ni_2_/rGO, and Pd_8_Ni_2_/rGO-POM nanomaterials for ORR in O_2_-saturated 0.1 M KOH electrolytes at a rotating speed of 1600 rpm (Fig. 5a). Pd_8_Ni_2_/rGO-POM demonstrated higher cathodic current density compared to other evaluated nanocatalysts. Noticeably, Pd_8_Ni_2_/rGO-POM revealed a low ORR onset potential (*E*_onset_) at −131 mV *vs.* Ag/AgCl, which is more positive relative to that of the Pd_8_Ni_2_/rGO catalysts (−152 mV), as well as that of the Pd/rGO catalyst (−178 mV) (Table 2), and comparable to that observed for Pt/C catalyst.^61^ The Pd_8_Ni_2_/rGO-POM displays the highest mass activity (MA) among all nanocatalysts (presented in Table 2). For instance, the MA values of Pd_8_Ni_2_/rGO-POM at kinetic region (−0.3 V) and diffusion-limited region (−0.8 V) are 18.16 and 28.75 mA mg_metal_^−1^, respectively, which are greater compared to those of the other investigated samples (Fig. 5b). These outcomes evidently unveil the boosted influence on oxygen reduction efficiencies using POM decorated on Pd_8_Ni_2_/rGO catalyst.

**Table 2:** Electro-chemical properties of the Pd/rGO, Pd_8_Ni_2_/rGO, and Pd_8_Ni_2_/rGO-POM electro-catalysts in 0.1 M KOH

Electro-catalyst	*E*_onset_ (mV *vs.* Ag/AgCl)	MA at −0.3 V (mA mg_metal_^−1^)	Tafel slope (mV dec^−1^)
Pd/rGO	−178	14.36	63.6
Pd_8_Ni_2_/rGO	−152	16.74	61.5
Pd_8_Ni_2_/rGO-POM	−131	18.16	59.7

Tafel analysis is very valuable in evaluating the electro-chemical kinetics of the oxygen reduction process. Fig. 5c depicts the Tafel plots of prepared materials in the low current intensity region. In general, lower Tafel slope values are the indication of better catalytic performance towards ORR. Among the studied nanocatalysts, Pd_8_Ni_2_/rGO-POM has the lowest Tafel slope of 59.8 mV dec^−1^ at the low current intensity area (*E*/V ≥ ∼−0.25 V), as shown in Fig. 5c and Table 2. When the Pd–Ni alloyed nanocatalyst is decorated with the POMs, the Tafel slope value can be diminished, suggesting that the existence of POM is beneficial to the improvement of ORR kinetics. Remarkably, the value of the Tafel slope for Pd_8_Ni_2_/rGO-POM showed excellent agreement with the typical values achieved for carbon-supported Pt catalyst in the literature.^62,63^ The close Tafel slope values of Pd_8_Ni_2_/rGO-POM and Pt/C manifest that the oxygen reduction kinetics is nearly similar for the two nanocatalysts.

**Fig. 6:**
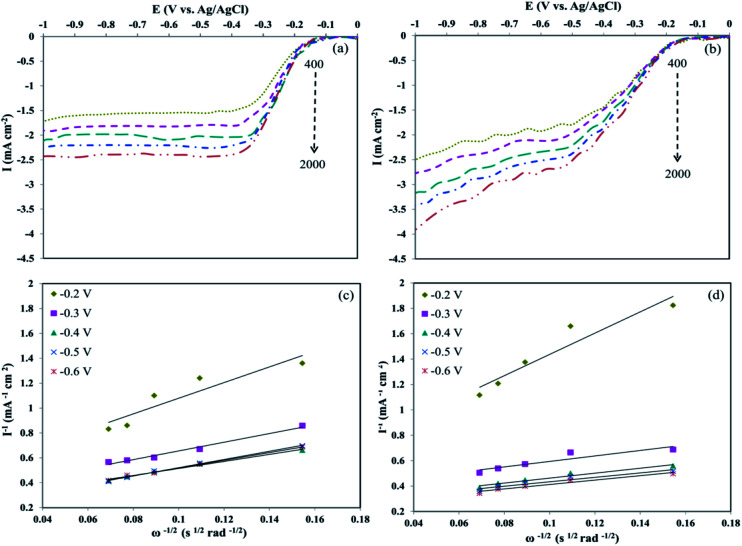
The RDE voltammograms of the Pd/rGO (a) and Pd_8_Ni_2_/rGO-POM (b) in O_2_-saturated 0.1 M KOH solution with a scan rate of 5 mV s^−1^ at various rotating speeds (rpm). The corresponding Koutecky–Levich plots of the Pd/rGO (c) and Pd_8_Ni_2_/rGO-POM (d) derived from RDE at different potentials.

Furthermore, LSVs were measured on the RDE for Pd/rGO and Pd_8_Ni_2_/rGO-POM with 400–2000 rpm at 5 mV s^−1^ scan rate (Fig. 6a and b). Fig. 6a and b show the RDE voltammograms of Pd/rGO and Pd_8_Ni_2_/rGO-POM with the achieved oxygen reduction currents. The increment in the currents was observed with the increment in the rotating rate, recommending the greater diffusion of oxygen to the surface of nanocatalyst directing to improved oxygen reduction *via* maximal transferred electron numbers. The cathodic current densities for Pd_8_Ni_2_/rGO-POM were higher than those for Pd/rGO under identical rotating rates.

The obtained findings highlight the importance of the POM adsorption on the rGO surfaces with well-distributed Pd–Ni alloyed nanoparticles. On the base of RDE experiments, the number of the electrons transferred during the oxygen reduction procedure was estimated by the Koutecky–Levich (K–L) equation as defined below:^64^1
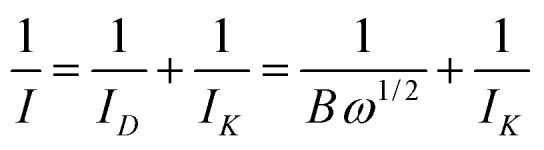

2 *ω* = 0.62*nF*(*D*_0_)^2/3^*C*_0_*υ*^−1/6^

where *I* is the acquired current density, *I*_K_ and *I*_D_ are the kinetic and diffusion-limited current density, respectively; *ω* is the electrode rotation rate (rad s^−1^), *n* is the number of electrons transferred in the oxygen reduction, *F* is the Faraday's constant (96 485 C mol^−1^), *D*_0_ represents the diffusion coefficient of dissolved O_2_ (1.9 × 10^−5^ cm^2^ s^−1^), *C*_0_ expresses the concentration of dissolved O_2_ in the electrolyte (1.2 × 10^−3^ M), and υ is the kinematic viscosity of the solution (1 × 10^−2^ cm^2^ s^−1^).^13,65,66^

Fig. 6c and d illustrate the particular K–L plots of Pd/rGO and Pd_8_Ni_2_/rGO-POM at selected electrode potentials drafted from the LSV curves. It evidently presented that the plots between *I*^−1^*vs. ω*^−1/2^ at diverse potentials for Pd_8_Ni_2_/rGO-POM are more linear compared to those for Pd/rGO, which signified that the reduction of oxygen on the surface of the Pd_8_Ni_2_/rGO-POM sample follows the first-order reaction kinetics.^67,68^ Based on eqn (1) and (2), the transferred electron numbers (*n*) on the studied catalysts can be assessed by using the measured slope of those K–L plots. In calculated potential ranges, the average of *n* values attained for catalyzing oxygen reduction on the Pd_8_Ni_2_/rGO-POM is around 3.9, which is near those on the carbon-supported Pt electro-catalyst reported in the literature.^11,69,70^ This experimental result discloses that the nearly 4e^−^ electron transfer mechanism is the predominant route for ORR catalysis on the Pd_8_Ni_2_/rGO-POM catalyst in alkaline solution.

#### 3.2.2. Chronoamperometry (CA)

**Fig. 7:**
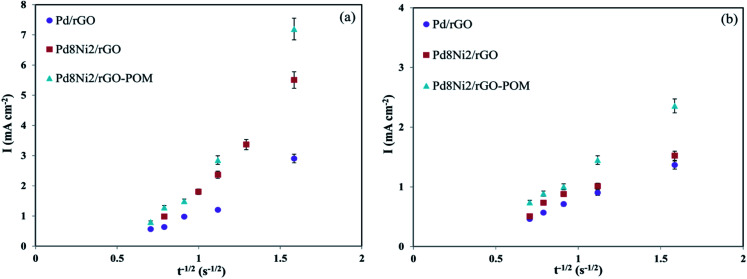
Plots of *I vs. t*^−1/2^ for the Pd/rGO, Pd_8_Ni_2_/rGO, and Pd_8_Ni_2_/rGO-POM electro-catalysts in O_2_-saturated 0.1 M KOH (a) and 0.1 M KOH containing 1 M methanol (b).

The chronoamperometric tests were utilized to examine the permeability of oxygen at the surface of fabricated electro-catalysts. The corresponding Cottrell plots (*I vs. t*^−1/2^) of prepared nanocatalysts in 0.1 M KOH solutions are revealed in Fig. 7a. The oxygen permeability values (*D*_b_^1/2^*c*_b_) attained from the slope of those *I vs. t*^−1/2^ plots based on the modified Cottrell equation^71^ are reported in Table 3. As evidently seen in Fig. 7a and Table 3, the Pd_8_Ni_2_/rGO-POM possesses a greater oxygen permeability value comparing to other evaluated samples, and this observation verifies its boosted ORR electro-catalytic capability. It is inferred that the easier diffusion of oxygen on the Pd_8_Ni_2_/rGO-POM surface can be assigned to the effect of alloying Ni with Pd, unique porous structures of rGO substrate, along with POM modification of alloyed catalyst.

**Table 3:** Oxygen permeabilities of the Pd/rGO, Pd_8_Ni_2_/rGO, and Pd_8_Ni_2_/rGO-POM electro-catalysts in the absence and presence of 1 M methanol

Electro-catalyst	In the absence of methanol *D*_b_^1/2^*c*_b_ (mol cm^−2^ s^−1/2^)	In the presence of 1 M methanol *D*_b_^1/2^*c*_b_ (mol cm^−2^ s^−1/2^)
Pd/rGO	1.33 × 10^−8^	5.41 × 10^−9^
Pd_8_Ni_2_/rGO	2.71 × 10^−8^	6.59 × 10^−9^
Pd_8_Ni_2_/rGO-POM	3.79 × 10^−8^	8.72 × 10^−9^

The chronoamperometric analyses of synthesized nanomaterials were also conducted to determine their oxygen permeability in 0.1 M KOH solutions containing 1 M methanol (Fig. 7b). The oxygen permeabilities for the resultant nanocatalysts are presented in Table 3. It is noticeably observed that the modification of the rGO-supported Pd_8_Ni_2_ catalyst with POM can give rise to larger values of oxygen permeability relative to those of other studied samples in alkaline media saturated with methanol. For the prepared nanocatalysts, it is evident that with the existence of 1 M methanol in the electrolyte, the permeabilities of oxygen are inferior to those without the addition of methanol. The inferior oxygen permeabilities of electro-catalysts in methanol-containing environments as compared with alkaline environments could be due to the accumulation of reactive positions for ORR by adsorbed methanol intermediates in the former environments. Remarkably, the chronoamperometric findings are consistent with the previously obtained experimental results in this study.

#### 3.2.3. Methanol resistance and durability tests

**Fig. 8:**
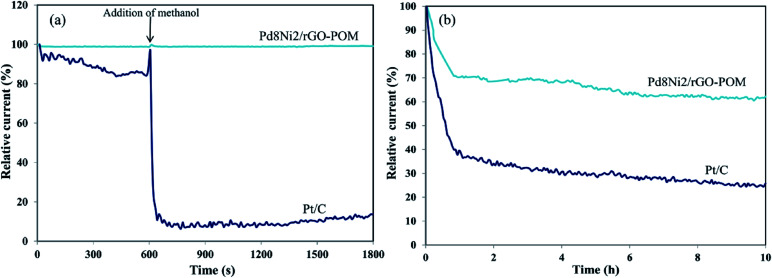
Methanol resistance tests of the Pd_8_Ni_2_/rGO-POM and commercial Pt/C upon adding 3 M methanol after 600 s in O_2_-saturated 0.1 M KOH solution at −0.4 V *vs.* Ag/AgCl (a). Current–time (*i*–*t*) durability measurements of the Pd_8_Ni_2_/rGO-POM and commercial Pt/C in O_2_-saturated 0.1 M KOH solution at −0.4 V *vs.* Ag/AgCl for 10 h (b).

The plausible crossover influence and long-term durability of nanocatalysts are considered as pivotal aspects for practicable implementation in the fuel cell systems.^72,73^ The methanol fuels at the anodic reservoir consistently pass across the membrane to the cathodic catalyst, which may lead to blocking the electrode potential equilibrium, thus diminishing the catalytic performance in fuel cells. The resistance to methanol crossover of Pt/C and Pd_8_Ni_2_/rGO-POM was assessed using the chronoamperometric response at −0.4 V *vs.* Ag/AgCl in O_2_-saturated 0.1 M KOH solutions (Fig. 8a). As indicated in Fig. 8a, when methanol (3 M) at 600 s was injected into the 0.1 M KOH solution, the oxygen reduction performance for Pt/C electro-catalyst presents a dramatic decrease in current density, proposing the existence of the methanol oxidation. Conversely, Pd_8_Ni_2_/rGO-POM recovers rapidly after the methanol injection. Therefore, the excellent resistance to methanol crossover and desired selectivity towards the reduction of oxygen make the prepared Pd_8_Ni_2_/rGO-POM nanocatalyst an encouraging candidate for the cathodic electrodes in DMFCs.

Moreover, the long-term durability of the Pd_8_Ni_2_/rGO-POM nanocatalyst towards oxygen reduction was tested using the chronoamperometry experiments in O_2_-saturated 0.1 M KOH at −0.4 V *vs.* Ag/AgCl, as displayed in Fig. 8b. During the chronoamperometry analysis, Pd_8_Ni_2_/rGO-POM exhibited greater durability, with almost 62.3% electro-catalytic ability retention after 10 h in alkaline condition, compared to that for carbon-supported Pt catalyst (just 25.8% retention). The improved durability can be associated with well-distributed Pd–Ni nanoparticles on the rGO substrate and POM modification of the Pd_8_Ni_2_/rGO sample. This experimental outcome specifies that the fabricated Pd_8_Ni_2_/rGO-POM nanocatalyst possesses superior durability for long-term usage as cathodic materials in alkaline fuel cell technologies.

On the basis of the above observations, the excellent ORR catalytic capability and methanol resistance of Pd_8_Ni_2_/rGO-POM could stem from several aspects. First, rGO, as a supporting material, improves electronic conductivity and mass transport capability. As well, the incorporation of Ni to Pd further enhances the catalytic activity and durability owing to the compressive strain and electronic ligand effects. When Ni is introduced into the Pd structure to make Pd–Ni alloy catalysts, the lattice of Pd is contracted, giving rise to the downshifted d-band positions and the decreased oxygen-binding strength for Pd. The electronic constructions of Pd can also be amended by the electron coupling between Pd and Ni to reach enhanced oxygen reduction performance. Second, the porous nanostructures and modifications in the electronic features, originating from the interplay between rGO and POM, offer much more points to evenly disperse nanosized Pd–Ni particles, available pathways for the permeation of oxygen during the ORR process and at the same time hamper the adsorption of methanol on reactive positions. Third, a synergistic effect of Pd–Ni, POM, and rGO is a benefit to promote the catalytic performances of fabricated catalysts.^27,40,46^ Thereby, the Pd_8_Ni_2_/rGO-POM nanomaterial is anticipated to be employed as a novel candidate to develop efficient oxygen reduction nanocatalysts for DMFCs.

## 4. Conclusions

In this work, a novel Pd–Ni/rGO-POM electro-catalyst was efficiently fabricated using the impregnation technique towards the ORR. The physical and morphological characteristics of prepared samples were studied by XRD, FT-IR, Raman, ICP-OES, FESEM-EDX, and TEM analyses. The outcomes verified the successful formation of nanocatalysts. The electro-chemical properties of fabricated nanocatalysts towards ORR were examined using CV, RDE, and CA. The Pd_8_Ni_2_/rGO-POM demonstrated outstanding oxygen reduction ability under alkaline conditions, which could be attributed to the incorporation of Ni metallic elements with Pd, the deposition of Pd_8_Ni_2_ alloyed particles on the rGO support, as well as the decoration of Pd_8_Ni_2_/rGO alloy nanocatalyst with POM. The Pd_8_Ni_2_/rGO-POM sample indicated impressive ORR electro-catalytic activity with a smaller Tafel slope, higher oxygen permeability, and a dominant 4-electron oxygen reduction process. In comparison with the commercially available Pt/C catalyst, the resistance to the crossover of methanol and long-term durability were also considerably enhanced. The Pd_8_Ni_2_/rGO-POM material reported here exhibits the promising prospect to be a favorable cathode electro-catalyst for DMFCs.

## Author contributions

Fereshteh Dehghani Sanij: conceptualization, investigation, methodology, formal analysis, writing – original draft. Prabhuraj Balakrishnan: investigation, writing – review & editing. Huaneng Su: methodology. Lindiwe Khotseng: methodology, writing – review & editing. Qian Xu: funding acquisition, supervision, writing – review & editing.

## Conflicts of interest

There are no conflicts to declare.

## Supplementary Material
